# Cardiovascular risk assessment in patients with a severe mental illness: a systematic review and meta-analysis

**DOI:** 10.1186/s12888-016-0833-6

**Published:** 2016-05-12

**Authors:** Quintí Foguet-Boreu, Maria Isabel Fernandez San Martin, Gemma Flores Mateo, Edurne Zabaleta del Olmo, Luís Ayerbe García-Morzon, Maria Perez-Piñar López, Luis Miguel Martin-López, Javier Montes Hidalgo, Concepción Violán

**Affiliations:** Institut Universitari d’Investigació en Atenció Primària Jordi Gol (IDIAP Jordi Gol), Universitat Autònoma de Barcelona, Gran Via Corts Catalanes, 587 àtic, 08007 Barcelona, Spain; Department of Medical Sciences, School of Medicine, University of Girona, Emili Grahit, 77, 17071 Girona, Spain; Técnica de Salud ICS, Unitat Docent AFiC, Sardenya, 375, Entl., 08025 Barcelona, Spain; Faculty of Nursing, University of Girona, Emili Grahit, 77, 17071 Girona, Spain; The Westborough Road Health Centre, 258 Westborough Road, Westcliff-on-Sea, SS0 9PT United Kingdom; Centre of Primary Care and Public Health, Queen Mary University of London, Yvone Carter Building 58 Tuner Street, E1 2AB London, United Kingdom; Departamento de Psiquiatría y Medicina Legal, Instituto de Neuropsiquiatría y Adicciones (INAD), Hospital del Mar Parc de Salut Mar., Universidad Autónoma de Barcelona, Passeig Marítim 25-29, 08003 Barcelona, Spain; Gimbernat School of Nursing, Universitat Autònoma de Barcelona, Avinguda de la Generalitat, 202-206, Sant Cugat del Vallès, 08174 Barcelona, Spain

**Keywords:** Cardiovascular risk, Severe mental illness, Depressive disorder, Bipolar disorder, Schizophrenia, Systematic review

## Abstract

**Background:**

Cardiovascular risk (CVR) has been observed to be higher in patients with severe mental illness (SMI) than in the general population. However, some studies suggest that CVR is not equally increased in different subgroups of SMI. The purposes of this review are to summarise CVR scores of SMI patients and to determine the differences in CVR between patients with different SMIs and between SMI patients and the control-population.

**Methods:**

MEDLINE (via PubMed) was searched for literature published through August 28, 2014, followed by a snowball search in the Web of Science. Observational and experimental studies that reported CVR assessments in SMI patients using validated tools were included. The risk of bias was reported using STROBE and CONSORT criteria. Pooled continuous data were expressed as standardized mean differences (SMD) with 95 % confidence intervals (CI). Two reviewers independently selected studies, extracted data and assessed methodological quality.

**Results:**

A total of 3,608 articles were identified, of which 67 full text papers were assessed for eligibility and 35 were finally included in our review, in which 12,179 psychiatric patients and 225,951 comparative patients had been assessed. The most frequent diagnoses were schizophrenia and related diagnoses (45.7 %), depressive disorders (14.7 %), SMI (11.4 %) and bipolar disorders (8.6 %). The most frequent CVR assessment tool used was the Framingham risk score. Subgroups analysis showed a higher CVR in schizophrenia than in depressive disorder or in studies that included patients with multiple psychiatric diagnoses (SMD: 0.63, 0.03, and 0.02, respectively).

Six studies were included in the meta-analysis. Total overall CVR did not differ between SMI patients and controls (SMD: 0.35 [95 % CI:−0.02 to 0.71], *p* = 0.06); high heterogeneity was observed (*I*^2^ = 93 %; *p* < 0.001).

**Conclusions:**

The summary of results from studies that assessed CVR using validated tools in SMI patients did not find sufficient data (except for limited evidence associated with schizophrenia) to permit any clear conclusions about increased CVR in this group of patients compared to the general population.

The systematic review is registered in PROSPERO: CRD42013003898.

**Electronic supplementary material:**

The online version of this article (doi:10.1186/s12888-016-0833-6) contains supplementary material, which is available to authorized users.

## Background

Cardiovascular disease is the leading cause of overall mortality, accounting for 24 % of deaths worldwide, while psychiatric diseases, led by major depressive disorder, are considered the eleventh most burdensome disease globally, with an increasing effect on overall mortality [1, 2].

Criteria for the definition of severe mental illness (SMI) differ, with some authors applying a narrow definition based on psychosis [3] and others also including a set of nosological entities of different types and clinical symptoms but with several common diagnostic criteria: severity, persistence over time (2 years or more), and a tendency toward clinical deterioration and difficulties in social and occupational function [4, 5].

It has been reported that cardiovascular risk (CVR) is higher in patients with SMI [6]. Studies in patients with bipolar disorder and schizophrenia indicate that they have a higher CVR than in the general population [7]. In patients with schizophrenia, the most prevalent CVR factors are hyperlipidaemia (61 %), smoking (55 %), obesity (41 %), diabetes (19 %) and hypertension (17 %)) [8]. Risk of metabolic syndrome is also higher among patients with schizophrenia and bipolar disorder [9]. Moreover, patients with anxiety and major depression have higher prevalence of hypertension compared to groups of similar age from the general population [10, 11].

Several factors may contribute to this raised CVR among patients with SMI, including unhealthy behaviours, difficulties in communication, barriers to medical care, poor treatment adherence and social deprivation [12]. Patients with SMI often receive fragmented medical care and fewer preventive measures, which leads to higher levels of underdiagnosis and lower rates of disease control [13]. Furthermore, antipsychotic drugs, antidepressants, and mood-stabilizing drugs have deleterious side effects, including important cardiometabolic consequences [14–16].

However, to date no systematic analysis has investigated whether CVR is increased equally in all patients with SMI, making it difficult to design and implement effective, feasible, evidence-based interventions for CVR management in these patients. A summary of the observations about CVR in the different subgroups of patients with SMI would provide a better epidemiological description of the problem, inform more effective clinical and preventive strategies and help in the design of further studies.

The major aim of this review was to summarize the available evidence of CVR scores in patients with SMI. Furthermore, this review attempted to determine whether CVR differs between subgroups of SMI patients and compare the CVR between patients with SMI and the general or non-psychiatric population.

## Methods

The Meta-analysis Of Observational Studies in Epidemiology (MOOSE) criteria were used to undertake this review and meta-analysis [17], together with Preferred Reporting Items for Systematic Reviews and Meta-Analyses (PRISMA) [18]. We conducted a systematic review of studies that reported CVR in patients with SMI.

### Eligibility criteria

We included studies that reported CVR scores in patients with SMI. The following 10 diagnoses were included in the search strategy (Table 1): schizophrenic disorders, schizotypal disorders, persistent delirious disorders, induced delirious disorders, schizoaffective disorders, other non-organic psychotic disorders, bipolar disorder, serious depressive episode with psychotic symptoms, recurrent serious depressive disorders, and compulsive obsessive disorder [5].

**Table 1:** Search strategies for the electronic databases (data retrieved August 28, 2014)

Database	Search Strategy	References
PubMed	("Psychotic Disorders"[Mesh] OR "Bipolar Disorder"[Mesh] OR "Schizophrenia"[Mesh] OR psychotic OR psychosis OR psychoses OR schizo* OR bipolar OR manic OR mania OR delirious OR depress* OR obsessive-compulsive OR "obsessive compulsive" OR “compulsive obsessive” OR OCD OR agoraphob* OR panic OR phobia OR phobic OR melanchol* OR neurosis OR neurotic OR neuroses OR conversion disorder* OR “Mental Disorders” OR “severe mental”) AND (cardiovascular OR “Cardiovascular diseases” OR CVD) AND (risk score* OR risk chart* OR “risk prediction” OR risk check* OR “risk assessment” OR “risk evaluation” OR “risk calculator” OR risk-estimation OR “risk estimation” OR "year risk" OR “year CVD risk” OR Framingham OR “SCORE risk” OR SCORE chart* OR SCORE table*OR “Systematic Coronary Risk Evaluation” OR “REGICOR” OR “REGICOR table”* OR ASSIGN OR QRISK OR PROCAM OR WHO/ISH)	653
Web of Science	Snowballing: references cited in the eligible papers (forward), and references citing the eligible papers (backward)	2,955

We included observational and experimental studies that applied validated CVR tools, including Framingham risk score (FRS) with its subtypes of scores (cardiovascular disease (CVD), cardiovascular heart disease (CHD), Myocardial infarction (MI) and the Systematic Coronary Risk Evaluation (SCORE). If the studies reported data on other CVR scores not described above, these were also included.

We excluded articles that were based on first episodes of SMI, different reports from the same population (selecting the study with the most recent publication date or the largest sample size), papers reporting diagnoses based on symptoms, and studies referring to one or two psychotropic drugs.

### Search strategy

We conducted a systematic search in PubMed using a combination of MESH and free text terms (Table 1). We searched from inception to the August 28, 2014. Based on the articles selected, we performed a snowball search in the Web of Science. We reviewed all the references (backward search) and the articles that cited the included papers (forward search). In addition, we added articles that were identified during the implementation of the review (hand searching). There were no language restrictions.

### Study selection

Two researchers (CVF and QFB) reviewed the titles and abstracts of all studies identified in the initial search and defined a list of full text articles to be assessed. Cases of discordance were resolved by consensus; when necessary, the full-text article was reviewed. We conducted a pilot test of the eligibility criteria on a sample of 15 articles. We used this test to clarify these criteria and ensure that they were applied consistently by all reviewers.

Primary outcome was the CVR assessed with any validated CVR tool.

### Data collection

We used a standardized data-collection form to record author and publication year, study design, country, setting, diagnosis, diagnostic criteria, number of participants and age in the psychiatric group and the comparative group (if applicable) and the objective of the study.

To assess the methodological quality of the studies, we used the Strengthening the Reporting of Observational Studies in Epidemiology Statement (STROBE) checklist for observational studies, with a maximum possible score of 24 [19], and the Consolidated Standards of Reporting Trials (CONSORT) for randomized trials, with a maximum possible score of 37 [20], giving one point for each item the article addressed.

Two reviewers assessed methodological quality and extracted the data independently. Discrepancies were resolved by consensus between the two reviewers (CVV and QFB) and by discussion with a third reviewer (MFS) as needed. Inter-rater agreement was 96 %.

### Statistical analysis

We analysed outcomes using Review Manager (RevMan, version 5.3). Pooled continuous data were expressed as standardized mean differences (SMD) with 95 % confidence intervals (CI). The effect size (ES) was categorized as small (<0.2), small to moderate (0.2–0.5), moderate to large (0.51–0.79), large (>0.79). Pooled SMD were estimated by using an inverse-variance-weighted random-effects model. Heterogeneity was quantified with the I^2^ statistic, which describes the proportion of the total between-study variability due to heterogeneity [21]. We used subgroup analysis to evaluate whether results differed according to the diagnosis (depressive disorder, schizophrenia vs psychiatric diagnoses), diagnosis criteria (non-specific (NE) vs DSM IV), study design (observational vs randomized control trial); and outcome (cardiovascular disease, coronary heart disease, stroke)

We assessed publication bias by using funnel plots. In sensitivity analysis, we assessed the relative influence of each study on the pooled estimate by omitting one study at time.

#### Protocol and registration

The initial protocol of the review was submitted to the International Prospective Register of Systematic Reviews (PROSPERO, http://www.crd.york.ac.uk/PROSPERO/). The definitive protocol included the modifications suggested by the PROSPERO reviewers. The registration number of systematic review is: CRD42013003898.

## Results

**Fig. 1:**
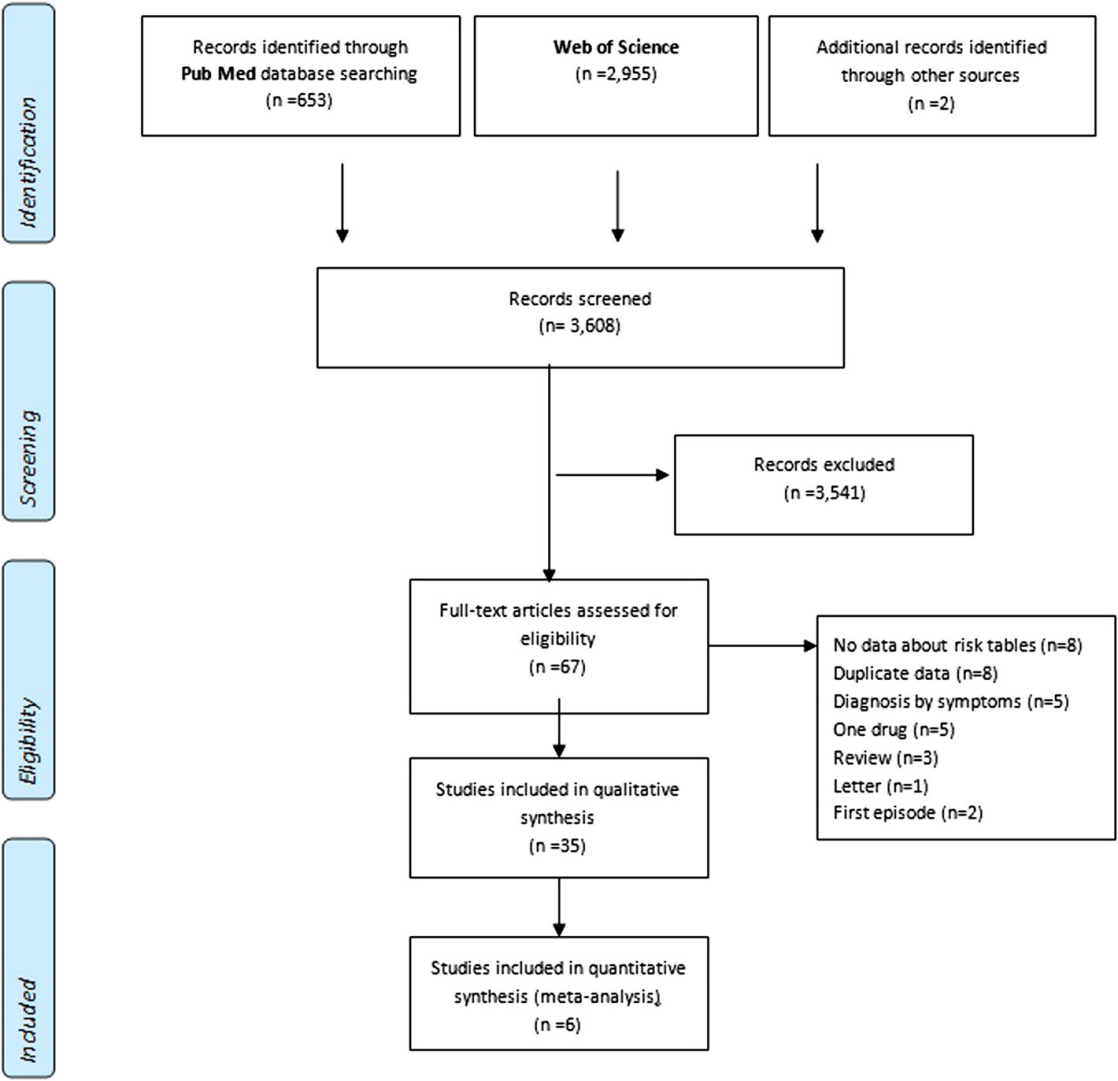
Flow diagram of article review process

The electronic and manual searches retrieved 3,608 articles, of which 67 full-text papers were assessed for eligibility and 35 studies were finally included in our review (Fig. 1), representing a total of 12,179 psychiatric patients and 225,951 controls. Sample size of psychiatric study groups ranged from 36 [22] to 1,942 [23] participants. Of the 35 studies, 19 studies in Eurasia (16 in Europe) and 16 studies were conducted in the Americas. The most common design was cross-sectional (22 studies); 8 studies were randomized controlled trials (RCT) and only 5 were case-control studies (Table 2). A 45.7 % of the studies were performed in secondary services exclusively and 31.4 % in the hospital setting. The most frequent diagnoses were schizophrenia and related diagnoses (45.7 %), depressive disorders (14.3 %), and bipolar disorders (8.6 %). Seven studies (20.0 %) included different psychiatric diagnoses and only 4 (11.4 %) showed data on SMI as a whole (Table 1).

**Table 2:** Characteristics of the studies included in the review

Author, year	Study design	Country	Setting	Diagnosis	Diagnostic criteria	Psychiatric Group						Comparative Group				
						Number	Age [mean(SD)]	FRS [mean (SD)]			SCORE [mean (SD)]	Number	Age [mean(SD)]	FRS [mean (SD)]		
								CVD	Stroke	CHD				CVD	Stroke	CHD
Acharya T, 2013 [43]	Retrospective cross-sectional	USA	Hospital	Depressive disorder	Not reported	1.136	60.1 (3.0)			By drug		472	61,4 (11.9)			17.1 (5.7)
Allan CL, 2011 [22]	Cross-sectional	UK	Primary & secondary care services	Depressive disorder	DSM IV	36	71.8 (7.7)		10.3 (7.6)			25	71.8 (7.3)		10.1 (7.7)	
Arango C, 2008 [44]	Cross-sectional	Spain	Secondary services	Schizophrenia & related disorders	DSM IV	1.452	40.7 (12.2)			6.8 (6.9)	0.9 (1.9)					
Bernardo M, 2009 [45]	Cross-sectional	Spain	Psychiatric hospital	Schizophrenia	DSM IV	733	37.8 (11.3)									
Cohn T, 2004 [37]	Cross-sectional	Canada	Psychiatric hospital & secondary services	Schizophrenia & related disorders	DSM IV	240	43.6 (1.3)					7,020	43.6 (1.3)			
Correll CU, 2006 [46]	Cross-sectional	USA	Psychiatric hospital	Psychiatric diagnosis	Not reported	367	42.9 (15.3)									
Correll CU, 2011 [47]	Cross-sectional	USA	Psychiatric hospital	Psychiatric diagnosis	Not reported	127	39.3 (14.9)			2.5 (4.2)						
Daumit GL, 2008 [48]	RCT	USA	Secondary services	Schizophrenia	DSM IV	1.125	40.7 (11.1)			8.5 (7.4)						
Dickerson FB, 2013 [49]	RCT	USA	Secondary services	SMI	DSM IV	291										
Druss BG, 2010 [50]	RCT	USA	Secondary services	SMI	Not reported	407										
Ferreira L, 2010 [51]	Case-control	Portugal	Secondary services	Schizophrenia	DSM IV	125	41.0 (11.0)					1,721	41.0 (12.0)			
Foguet-Boreu Q, 2013 [32]	Cross-sectional	Spain	Secondary services	SMI	Not reported	137	51.1 (12.9)									
Garcia-Portilla MP, 2009 [52]	Cross-sectional	Spain	Secondary services	Bipolar disorders	ICD10	194	46.6			7.6 (7.4)	1.8 (4.4)					
Goodrich DE, 2012 [53]	RCT	USA	Secondary services	Schizophrenia	Not reported	134	52.8 (9.9)									
Goff DC, 2005 [24]	Case-control	USA	Secondary services	Schizophrenia	DSM IV	689	40.4 (11.2)					687	40.4 (11.2)			6.5
Grover S, 2014 [54]	Cross-sectional	India	Hospital	Bipolar disorder	ICD10	105	39.6 (13.1)			3.4 (5.0)	1.7 (1.8)					
Hoffman BM, 2010 [55]	RCT	USA	H ospital	Depressive disorder	DSM IV	46	53.4 (7.0)	14.0 (9.0) Only males.								
Jin H, 2011 [56]	Cross-sectional	USA	Secondary services	With psychotic symptoms	DSM IV	179	63.1									
Mackin P, 2007 [25]	Case-control	UK	Secondary services	Psychiatric diagnosis	Not reported	90	45.7 (11.8)	11.3 (12.3)	1.7 (3.2)	9.3 (10.5)		92	43.5 (13.6)	6.8 (6.4)	1.0 (1.1)	4.7 (4.3)
Margari F, 2013 [26]	Cross-sectional	Italy	Psychiatric hospital	Psychiatric diagnosis	DSM IV	83	47.0 (9.0)	8.3 (5.8)				77	52.0 (8.6)	10.7 (5.9)		
McCreadie RG, 2003 [27]	Cross-sectional	UK	Secondary services	Schizophrenia	DSM IV	102	45.0 (13.0)			9.5 (7.6)						4.1 (4.0)
McLean G, 2014 [23]	Cross-sectional	UK	Primary care	Schizophrenia & related disorders	Read code	1.942						215,165				
Nurjono M, 2014 [57]	Cross-sectional	Singapore	Psychiatric hospital	Schizophrenia	DSM IV	64										
Osborn DP, 2006 [36]	Cross-sectional	UK	Primary care	SMI	Not reported	74						148				
Protopopova D, 2012 [58]	Cross-sectional	Czech Republic	Psychiatric hospital	Schizophrenia & pychoses	ICD10	129	36.0 (11.9)									
Ratliff JC, 2013 [28]	Case-control	USA	Secondary services	Schizophrenia & related disorders	DSM IV	115	47.5 (8.3)	10.7				197	47.7 (8.5)	8.5		
Said MA, 2012 [59]	Cross-sectional	Malaysia	Hospital	Schizophrenia	DSM IV	270				6.3 (5.6)						
Stroup TS, 2013 [60]	RCT	USA	Secondary services	Schizophrenia & related disorders.	Not reported	215	41.1 (11.1)			7.3 (5.7)						
Sicras-Mainar A, 2013 [61]	Cross-sectional	Spain	Primary, secondary & hospital care services	Schizophrenia & related disorders	DSM IV	705	48.2 (15.8)	11.9 (5.7)								
Slomka JM, 2012 [62]	RCT	USA	Secondary services	Bipolar disorder	Not reported	118	53.0 (9.9)	13.7 (10.0)								
Smith PJ, 2007 [63]	RCT	USA	Not reported	Depressive disorder	DSM IV	198	51.6 (7.5)			5.4 (3.2)						
Tay YH, 2013 [29]	Cross-sectional	China	Secondary services	Schizophrenia	DSM IV	83	36.2 (7.7)	4.7 (4.7)				243	34.6 (8.2)	3.1 (3.2)		
Taylor V, 2010 [64]	Case-control	Canada	Secondary services	Bipolar disorder & major depressive disorder	DSM IV	54	25.9 (7.0)					104				
Wysokiński A, 2012 [65]	Retrospective review	Poland	Psychiatric hospital	Psychotic disorder	ICD10	62	38.0 (12.4)	6.4 (7.2)	3.7 (2.8)	5.8 (6.1)						
Zuidersma M, 2015 [66]	Cross-sectional	Netherlands	Primary & secondary care services	Depressive disorder	DSM IV	352	70.7 (7.4)	5.8 (3.8)								

*Abbreviations*: *FRS* Framingham risk score, *CVD* cardiovascular disease, *CHD* cardiovascular heart disease, *MI* myocardial infarction, *SCORE* systematic coronary risk evaluation, *NE*: *SMI* severe mental illness, *DSM-IV* diagnostic and statistical manual of mental disorders, 4th Edition, *ICD-10* International classification of diseases, 10th revision, *RCT* randomized controlled trial

In 30 studies, methodological quality was evaluated with STROBE and most showed a high quality score (median 21.00, SD: 6.40). Five were evaluated with CONSORT and most had a low quality score (median 18.57, SD: 2.72). The STROBE evaluation revealed two main weaknesses: insufficient efforts to address potential sources of bias and sparse information for each variable of interest on the number of participants with missing data. The CONSORT weaknesses were the method used to generate the random allocation sequence and type of randomisation; details of any restriction (such as blocking and block size); and information about where the full trial protocol can be accessed, if available (Additional file 1: Appendix 1).

Of the 35 studies included, only 7 studies had control groups [22, 24–29]. These studies used different scores to evaluate CVR (Table 2). Three studies included only patients with schizophrenia and controls: two studies were based on FRS (CVD) scores: 10.7 vs. 8.5 *p* ≤ 0.01 [28] and 4.7 (4.7) vs. 3.1 (3.2), *p* = 0.002 [29] and one was based on FRS (CHD) scores: 8.6 (7.3) vs. 6.3 (6.0), *p* < 0.001 [24]. Two studies included psychiatric diagnoses and were based on FRS (CVD) scores: 11.3 (12.3) vs. 6.8 (6.4), *p* < 0.01 [25] and 8.3 (5.8) vs. 10.7 (5.9), *p* = 0.05 [26]. One study included depressive disorders: 10.3 (7.6) vs. 10.1 (7.7), *p* = 0.97 [22]. One study had insufficient data and was not included in the meta-analysis.

Table 3 synthesized the data about CVR scores found in studies by diagnosis groups of diseases. The CVR mean score assessed with FRS (CVD) in patients with depression ranged from 5.8 to 14.0, in patients with schizophrenia from 4.7 to 11.9, and was 13.7 in the only study of patients with bipolar disorder. Studies that addressed patients with SMI reported that CVR had been expressed in different forms (Table 3).

**Table 3:** Cardiovascular risk assessment by diagnostic group (depressive, bipolar, SMI and schizophrenia)

Diagnosis Groups	Author, year	Psychiatric Group				Notes
		FRS [mean (SD)]			SCORE [mean (SD)]	
		CVD	Stroke	CHD		
Bipolar disorder	Grover S, 2014 [54]			3.4 (5.0)	1.7 (1.8)	
	Slomka JM, 2012 [62]	13.7 (10.0)				
	Garcia-Portilla MP, 2009 [52]			7.6 (7.4)	1.8 (4.4)	
Depressive disorder	Acharya T, 2013 [43]					FRS (CHD) expressed by types of antidepressive medication groups.
	Allan CL, 2011 [22]		10.3 (7.6)			
	Hoffman BM, 2010 [55]	14.0 (9.0)				
	Smith PJ, 2007 [63]			3.2		
	Zuidersma M, 2015 [66]	5.8 (3.8)				
Schizophrenia	Bernardo M, 2009 [45]					SCORE: <1 %: 15.1 %; 1–4 %:68.8 %; 5–10 %: 6.1 %; 11–15 %:0.3 % and ≥15 %;0.1 %
	Daumit GL, 2008 [48]			8.5 (7.4)		
	Ferreira L, 2010 [51]					SCORE: no statistically significant difference between case and controls was observed.
	Goodrich DE, 2012 [53]					FRS (CVD): <10 %: 40.7 %, 10–20 %: 40.7 % and >20 %: 18.6 %
	Goff DC, 2005 [24]					FRS (CHD): In men: CATIE study: 9.4 (7.2); NHANES study: 7.0 (6.3) and in women: 6.3 (6.3) and 4.2 (4.5), respectively.
	McCreadie RG, 2003 [27]		4.1	9.6		
	Nurjono M, 2014 [57]					FRS (CVD): Participants in the highest quartile of serum BDNF had a 3.3 times increased in FRS over those in the lowest quartile.
	Said MA, 2012 [59]			6.3 (5.6)		31.5 % of patients in the metabolic syndrome group had a high/very high FRS (CHD) vs. 11 % in non-metabolic syndrome group (*p* < 0.001)
	Tay YH, 2013 [29]	4.7 (4.7)				
	Protopopova D, 2012 [58]					SCORE ≥ 5 %: 10 %
	Arango C, 2008 [44]			6.8 (6.9)	0.9 (1.9)	
	Cohn T, 2004 [37]					FRS (MI): 8.9 % in males, compared with control subjects (6.3) (*p* < 0.001) and 2.6 % females (vs. Control subjects 2.0 %) (*p* = 0.180).
	McLean G, 2014 [23]					Joint British Societies score: risk levels by age group and gender. Age was a major factor being identified as high risk (>20 %), with 79 % of those with schizophrenia aged 65–74 estimated at high risk compared with only 1.3 % of those aged 35–44.
	Ratliff JC, 2013 [28]	10.7				
	Stroup TS, 2013 [60]			7.3 (5.7)		
	Sicras-Mainar A, 2013 [61]	11.9 (5.7)				
SMI	Dickerson FB, 2013 [49]					FRS (CVD) in smokers 13.2 (11.9) and nonsmokers 7.4 (7.2)
	Druss BG, 2010 [50]					FRS (CHD): 6.9 for intervention and 9.8 for control group.
	Foguet-Boreu Q, 2013 [32]					FRS (REGICOR): high (≥10 %): 4.6 % and SCORE: high (≥5 %): 5.4 %
	Osborn DP, 2006 [36]					FRS (CHD): median: 5 % (IQR:2–12)
Psychiatric diagnoses	Wysokiński A, 2012 [65]	6.4 (7.2)	3.7 (2.8)	5.8 (6.1)		
	Correll CU, 2006 [46]					FRS (CHD): 8.29 (0.49) in men and 2.33 (0.52) in women.
	Jin H, 2011 [56]					FRS (CHD) was increased by 79 % in schizophrenia, 72 % in posttraumatic stress disorder and 61 % in mood disorder.
	Mackin P, 2007 [25]	11.3 (12.3)	1.7 (3.2)	9.3 (10.5)		
	Margari F, 2013 [26]	8.3 (5.8)				
	Correll CU, 2011 [47]			2.5 (4.2)		
	Taylor V, 2010 [64]					FRS (CHD) was lower for patients at baseline and follow-up, but increased across the follow-up period (2-years). Women patients showed an increase risk for CHD over time, and men did not.

Data are expressed as mean (SD), unless otherwise stated

*Abbreviations*: *FRS* Framingham risk score, *CVD* cardiovascular disease, *CHD* cardiovascular heart disease, *MI* myocardial infarction, *SCORE* systematic coronary risk evaluation, *SMI* severe mental illness, *PTSD* posttraumatic stress disorder

Subgroup analysis was performed in six studies (3 involving schizophrenia, 1 depressive disorder and 2 psychiatric diagnoses in general). Higher CVR was observed in patients with schizophrenia than in those with depressive disorder or general psychiatric diagnosis, with a pooled SMD (95 % CI) as follows: 0.63 (0.16, 1.09), 0.03 (−0.48, 0.54), and 0.02 (−0.82, 0.86), respectively (Table 4). The sensitivity analysis omitted one study at a time, showing a pooled SMD ranging from 0.19 to 0.50. Funnel plots did not suggest any publication bias (Additional file 2: Appendix 2).

**Table 4:** Stratified pooled standardized mean differences for cardiovascular risk assessment

	Number of studies	SMD	*I* ^2^
		(95 % CI)	
Diagnosis			
Depressive disorder	1	0.03 (−0.48, 0.54)	0 %
Schizophrenia	3	0.63 (0.16, 1.09)	94 %
Psychiatric diagnosis	2	0.02 (−0.82, 0.86)	92 %
Criteria for diagnosis			
NE	1	0.35 (0.24, 0.45)	84 %
DSM IV	5	0.32 (−0.11, 0.75)	94 %
Study design			
Observational study	5	0.34 (−0.21, 0.88)	94 %
RCT	1	0.35 (0.24, 0.45)	0 %
Outcome			
CVD total	4	0.40 (−0.44, 1.02)	95 %
CHD	1	0.35 (0.24, 0.45)	0 %
Stroke	1	0.03 (−0.48, 0.54)	0 %

*Abbreviations*: *SMD* standardized mean difference, *CI* confidence interval, *NE* non-specific, *DSM IV* statistical manual of mental disorders, *RCT* randomized controlled trial, *CVD* cardiovascular disease, *CHD* coronary heart disease

**Fig. 2:**
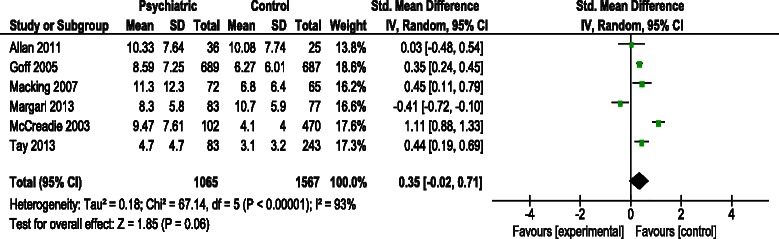
Summary of forest plot. Standardized mean differences between psychiatric group and comparative group

Six studies that included 1,065 people with SMI who had a CVR assessment and 1,567 people without SMI were included in the meta-analysis [22, 24–27, 29]. The total overall CVR between the psychiatric group and control group showed a SMD of 0.35 (95 % CI:−0.02–0.71, *P* = 0.06) with significantly high heterogeneity (*I*^2^ = 93 %; *p* < 0.001) (Fig. 2).

## Discussion

Data from studies that reported CVR scores did not support higher risk in patients with SMI than in the control population. Subgroup analysis showed a higher CVR associated with schizophrenia than with other SMIs. Only in patients with schizophrenia was there some evidence of higher CVR scores than in the control population.

To date it has been widely accepted that the prevalence of modifiable risk factors was increased in patients with SMI [30]. Nonetheless, several authors have suggested that not all risk factors were equally increased in these patients. A number of studies have found that smoking and diabetes rates were higher in the SMI population than in the reference population [31], while others observed that hypertension was not increased among SMI patients [31–33]. Conversely, other authors did not detect significant differences in CVR factors between participants with and without SMI [34]. This could be the result of publication bias affecting CVR studies and therefore affecting CVR assessment. Chapman et al., in a meta-analysis of 42 studies on smoking in patients with schizophrenia, revealed that studies reporting low prevalence of this risk factor are cited less often than those reporting higher prevalence in this population [35].

Numerous previous studies have noted the importance of CVR factors in patients with SMI, but only a few of them incorporated CVR evaluation in the last 12 years. No other systematic review has been found in the literature on this topic. However, Osborn et al., in a systematic review which objective was to determine the relative risk of some CVR factors in people with SMIs, synthesized data about some studies that reported 10 year cardiovascular risk scores [31]. One controlled community study, including 74 SMI patients found that excess CVR scores showed that participants with SMI had higher FRS (CHD) than participants without SMI (median 10-year risk: 5 vs. 4 %) [36]. Another study, including 84 schizophrenic patients showed a significant increase of CVR only in males based on FRS (CHD) (10.4 vs. 6.4 %) [27]. And the last, involves 240 patients schizophrenia and schizoaffective disorders showed also an increased risk based on FRS (MI) score only in male patients compared to controls (8.9 vs. 6.3 %) [37]. Our review also showed that schizophrenia is the group that have more evidence of higher CVR than control groups and is consistent with other studies [38, 39].

However, the discrepancy between data showing higher CVR in SMI and the CVR assessment obtained in our review raises some questions. The tools to measure CVR that have been validated for general population may not apply to patients with SMI. In this sense, Osborn et al. proposed a CVR prediction model for this population [40]. In addition to the usual predictors, this model also included social deprivation, heavy alcohol use, SMI diagnosis, and prescriptions for antidepressants and antipsychotics [40]. Another key point is the influence of the prescribed medications on CVR. There is strong evidence that antipsychotic drugs, and to a more restricted degree antidepressants and mood stabilizers, are associated with an increased risk for several physical diseases, including obesity, dyslipidaemia, diabetes mellitus and so on [41]. Furthermore, unclear benefits of different kinds of antipsychotics (first vs. second-generation antipsychotics) have been reported [42], despite the superior efficacy and greater treatment persistence attributed to second-generation antipsychotics.

Our analysis showed that schizophrenia is the group at highest risk, in consonance with other studies that showed an increased risk in patients with schizophrenia and depression, compared to other SMIs [41, 42]. Of the three studies of schizophrenic patients included in the forest plot summary, McCreadie et al. [27] clearly had the highest SMD. This difference may be explained by the inclusion of older patients with a longer history of illness compared to the other two studies [24, 29].

### Strengths and weaknesses of this review

The major strength of this study is that it is the first review to focus on CVR assessments in patients with SMI. The search identified a large number of studies (67) that showed CVR data. Osborn et al. centred their attention on studies of CVR factors and also showed results of CVR assessments provided by 4 studies, three of which included only schizophrenia and related disorders; one study also included non-affective chronic psychotic illness [31].

Our study also has a number of limitations to be taken into account. We only searched a single data source, although this limitation was countered by an extensive manual search (snowball method). Furthermore, a large number of studies had no control group, making it impossible to include them in the meta-analysis. Of the 7 studies with control groups [22, 24–29], only 6 had sufficient data for inclusion in the meta-analysis and the heterogeneity of data synthesis was considerable (I^2^ > 75 %). Therefore, all the conclusions of the meta-analysis should be considered with caution. The variability of the studies included in the meta-analysis could be attributed to the ages of the participants, the diagnoses included, and differences in study design.

### Implications for future research

Further work is needed to establish whether patients with SMI have increased CVR compared to general population. More information on the type of CVR assessment used would help to establish a greater degree of accuracy on this question. A new risk assessment approach may be needed in future studies in order to include other relevant factors (obesity, psychotropic drugs and social deprivation) [38]. In addition, a discussion is needed to reach consensus on an operational definition of SMI that can be applied for research purposes.

## Conclusions

A review of literature reporting CVR assessment in patients with SMI did not find sufficient evidence to determine whether or not there is a higher risk in these patients relative to the reference population. Subgroup analysis showed a higher CVR in patients with schizophrenia compared to those with depressive disorder or a psychiatric diagnosis. Only in patients with schizophrenia was there some evidence of higher CVR scores than in the control population. Instead of the generalized idea that SMI is associated with increased CVR, it is important to consider the complexity of summarizing the data because there is no universal definition of SMI or standard methods to describe CVR in this population. Further work is needed to elucidate whether new CVR charts that incorporate intrinsic determinants (as the effect of psychotropic drugs or social deprivation) should be established for risk assessment in this population.

### Ethics and consent to participate

Not Applicable.

### Consent to publish

Not Applicable.

### Availability of data and materials

The data and materials used in this review are available on request.

## Additional files

Additional file 1: Appendix 1. Quality assessment of the observational studies retained in the Review (STROBE). Quality assessment of the clinical trials studies retained in the Review (CONSORT). (DOC 97 kb) Additional file 2: Appendix 2. Funnel plot. (JPG 26 kb)

## Abbreviations

CHD: cardiovascular heart disease
CVD: cardiovascular disease
CVR: cardiovascular risk
DSM-IV: diagnostic and statistical manual of mental disorders, 4th edition
FRS: Framingham risk score
ICD-10: International Classification of Diseases, 10th revision
MI: myocardial infarction
SCORE: systematic coronary risk evaluation
SMI: severe mental illness
WHO: World Health Organization
