# Supplementary material for: Cardiovascular risk assessment in patients with a severe mental illness: a systematic review and meta-analysis
Source: BMC Psychiatry. 2016 May 12;16:141. doi: 10.1186/s12888-016-0833-6 (PMC4866037; doi:10.1186/s12888-016-0833-6)
Supplement: Additional file 1: Appendix 1. — Quality assessment of the observational studies retained in the Review (STROBE). Quality assessment of the clinical trials studies retained in the Review (CONSORT). (DOC 97 kb) [file 12888_2016_833_MOESM1_ESM.doc]

**Supplemental data: Appendix 1**

1. Quality assessment of the observational studies retained in the Review (STROBE)

1. Quality assessment of the clinical trials studies retained in the Review (CONSORT)
