# Supplementary figures and images for: Cardiovascular risk assessment in patients with a severe mental illness: a systematic review and meta-analysis
Source: BMC Psychiatry. 2016 May 12;16:141. doi: 10.1186/s12888-016-0833-6 (PMC4866037; doi:10.1186/s12888-016-0833-6)

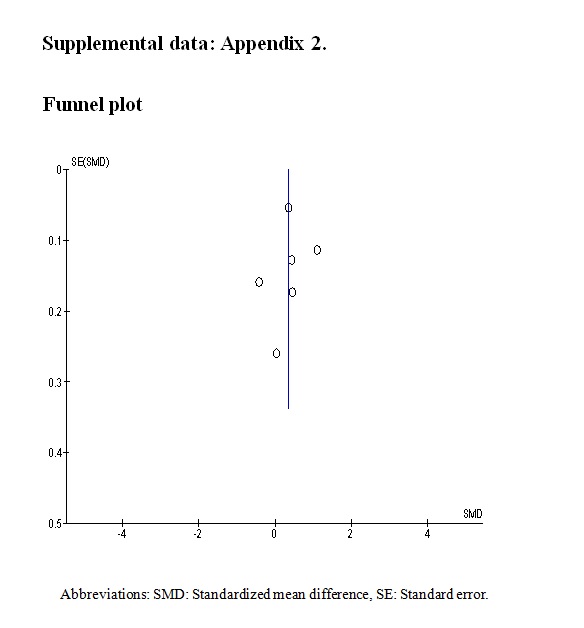

Supplement: Additional file 2: Appendix 2. — Funnel plot. (JPG 26 kb) [file 12888_2016_833_MOESM2_ESM.jpg]
